# Reweighting national survey data for small area behaviour estimates: modelling alcohol consumption in Local Authorities in England

**DOI:** 10.1186/s12963-019-0201-0

**Published:** 2020-01-02

**Authors:** Robert Pryce, Colin Angus, John Holmes, Duncan Gillespie, Penny Buykx, Petra Meier, Matt Hickman, Frank de Vocht, Alan Brennan

**Affiliations:** 1 School of Health and Related Research, University of Sheffield, 30 Regent Street, Sheffield, S1 4DA UK; 2 School of Humanities and Social Science, Newcastle University, Newcastle, New South Wales Australia; 3 Population Health Sciences, University of Bristol, Canynge Hall, 39 Whatley Road, Bristol, BS8 2PS UK

**Keywords:** Small area estimation, Reweighting, Alcohol

## Abstract

**Background:**

There are likely to be differences in alcohol consumption levels and patterns across local areas within a country, yet survey data is often collected at the national or sub-national/regional level and is not representative for small geographic areas.

**Methods:**

This paper presents a method for reweighting national survey data—the Health Survey for England—by combining survey and routine data to produce simulated locally representative survey data and provide statistics of alcohol consumption for each Local Authority in England.

**Results:**

We find a 2-fold difference in estimated mean alcohol consumption between the lightest and heaviest drinking Local Authorities, a 4.5-fold difference in abstention rates, and a 3.5-fold difference in harmful drinking. The method compares well to direct estimates from the data at regional level.

**Conclusions:**

The results have important policy implications in itself, but the reweighted data can also be used to model local policy effects. This method can also be used for other public health small area estimation where locally representative data are not available.

## Background

Recent estimates from the Global Burden of Disease study suggest that 17% of the total burden of ill health in England is due to behavioural risk factors and that there is significant variation across the country’s 9 regions in both the scale and pattern of associated harms [14]. These variations and those in the risky health behaviours are likely to be even greater at smaller levels of geography because predictors of both behaviour and harm—including sociodemographic characteristics [10, 18], availability of harmful commodities [1, 11, 17] and regional cultural differences [11, 18]—have been shown to vary markedly across such geographies. Set against this background, there has been increasing devolution of responsibility for public health policy decisions to Local Authorities in England, driving a need for local-level data on health behaviours and harms. This paper can provide locally specific evidence to target policies which effectively and cost-effectively reduce public health problems and associated health inequalities.

Whilst harm data are often available at local level from routinely collected records on deaths and hospital admissions, data on health behaviours usually come from government-funded large-scale surveys, which are representative only at the national, or some other large geographical, level. The implication of this is that, given the small samples, direct estimation of small area characteristics is not possible for each area, posing a challenge to policymakers wanting to know the pattern of health behaviours in their locality. This has further implications for modelling effects of policy on a small geographical scale. A common method for small area characteristics is to produce point estimates of a variable of interest, for example for smoking rates [19, 20], poverty [8] and multi-morbidities [13]. Alcohol consumption has previously been estimated at the local level, producing synthetic estimates of the proportion of the population who are abstainers and lower risk, increasing risk or higher risk drinkers within English Local Authorities [4].

The method we present in this paper goes beyond creating synthetic point estimates of alcohol consumption; \instead, it reweights individual-level survey data to make it representative of the local area’s sociodemographic characteristics and expected alcohol consumption. In this sense, it is similar to work by Twigg et al. [21]. The reweighted data can then be used to produce an estimate of the complete distribution of drinking in an area, and, by comparing across different reweighted datasets, demonstrate variation across areas. Our method and results are valuable, as understanding the distribution of drinking across the population of a given area is key to estimating both the overall and distributional effects of public health interventions in that area [12]. Detailed local estimates are also more informative to local policymakers seeking to identify the relative magnitude of public health problems and their distribution across society and the potential of policies they may enact to address these. Recent studies have sought to estimate the effects of such local policy approaches, for example on the impact of licencing restrictions [6, 7], and such investigations could be enhanced through data on local alcohol consumption patterns.

The purpose of this paper is to present a reweighting method that combines population characteristics with local area characteristics to estimate new weights that can make a national survey representative of the local population with reference to key characteristics, for example to create a synthetic “Health Survey for Sheffield” from the Health Survey for England. Clearly, this method is not limited to either alcohol (or even health-related behaviours) or Local Authority geography or England. It allows any survey which is representative at a large scale to be adjusted to be representative at a smaller scale. Therefore, the contribution of this work is threefold. First, we present a method of reweighting survey data to generate a locally representative version which is potentially useful for a variety of purposes. Second, in the calculation of new weights, we provide updated estimates of the proportion of the population drinking at different levels. The estimates compare well with direct comparison with the original data at region level. Third, we create a reweighted Health Survey for England for each of the 151 Upper Tier Local Authorities,^1^ which we can use for modelling local area policy effects.

## Methods

The reweighting method involves 3 steps. First, the probability of an individual belonging to one of seven alcohol consumption bands is estimated using statistical modelling of the Health Survey for England and adjusted for individual socio-demographic factors and for local area-level factors (in this case using Local Authority alcohol-attributable hospital admission rates and mortality rates). These probabilities are calculated for every combination of demographic characteristics and local factors. Second, the probabilities are then multiplied by the corresponding number of individuals within a Local Authority to provide estimates of the number of people in each of the seven alcohol consumption bands in the Local Authority’s population. These two steps are identical to the method employed by Beynon et al. [4]. The third step goes beyond small area point estimation by reweighting the survey data. This is done by dividing the number in the population with certain characteristics by the number of survey respondents with the same characteristics. This produces the reweighted survey that is locally representative and can be used directly for statistical analysis or incorporated into more complex modelling work to produce locally representative policy effect estimates.

**Fig. 1:**
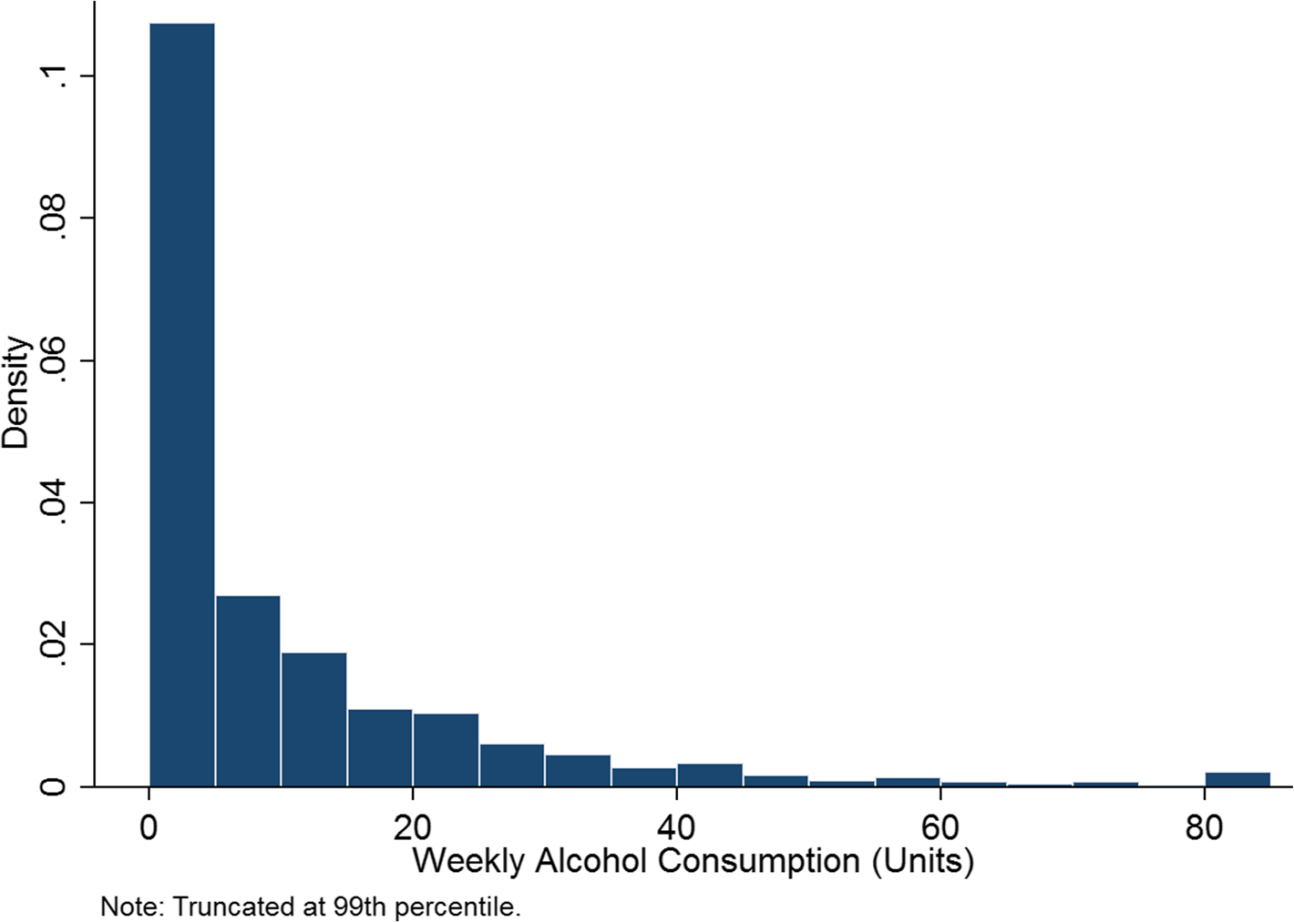
Alcohol consumption distribution from the Health Survey for England 2011–2013

The underlying dataset to be reweighted is the Health Survey for England (HSE), which is a nationally representative, repeated cross-sectional survey of roughly 8000 individuals in private households per year, covering health and health-related behaviours.^2^ The HSE contains information about each household member including age, sex, ethnicity and alcohol consumption. We also received information on the respondent’s Upper Tier Local Authority (UTLA) of residence. To avoid disclosure issues, UTLAs in London are either listed as inner or outer London. The HSE also provides survey weights to make the survey representative at the national level, but we do not use these to create the new, UTLA-level weights because they only correct for national-level sample representativeness. To create a large enough sample, the HSE data from 2011 to 2013 were pooled to give a sample of 25,086 adults aged 18 or over. This reduces to 24,685 for the final analysis because of missing information regarding ethnicity or alcohol consumption for 401 respondents. A sample size of 24,685 and a total of 151 UTLAs mean an average of just over 162 respondents in the survey per UTLA, meaning direct estimation of drinking patterns at the UTLA level would suffer from small sample problems. Respondents in the HSE are asked questions about their frequency of consuming, and typical consumption quantities, for several alcoholic beverages. This allows a total mean weekly alcohol consumption variable to be constructed which is measured in units of alcohol. A UK unit is 10 ml, or 8 g, of pure alcohol. The distribution of alcohol consumption in the HSE is shown in Fig. 1. Seventeen percent of the sample did not drink alcohol in the past year, and roughly three quarters of drinkers drink moderately (less than 14 units per week). Summary statistics of the sample are presented in Table 1.

**Table 1:** Summary statistics for Health Survey for England dataset (2011–2013 pooled)

Characteristic	*N*	%
Age band		
18–24	1805	7.31
25–34	3706	15.01
35–54	8753	35.46
55+	10,421	42.22
Sex		
Male	10,946	44.34
Female	13,739	55.66
Ethnicity		
White	22,015	89.18
Asian	1549	6.28
Other	1121	4.54
Index of multiple deprivation quintile		
1 (least deprived)	5367	21.74
2	4847	19.64
3	5217	21.13
4	4770	19.32
5 (most deprived)	4484	18.16
Mean weekly alcohol consumption *x* (units)		
0	4263	17.27
0 < *x* ≤ 10	12,357	50.06
10 < *x* ≤ 20	3700	14.99
20 < *x* ≤ 30	2049	8.30
30 < *x* ≤ 40	885	3.59
40 < *x* ≤ 50	612	2.48
50 < *x*50 < *x*	819	3.32

Each respondent’s weekly alcohol consumption is assigned to one of seven consumption bands (abstainer, 1–10 units, 11–20 units, 21–30 units, 31–40 units, 41–50 units, 51+ units per week) and the probability of drinking at each consumption band is estimated using a logistic regression to predict abstention and a multinomial logistic regression to predict positive consumption bands. The multinomial logistic regression is preferred to the ordered logistic regression because it allows the most flexibility and does not impose the proportional odds assumption.^3^ These are estimated as a function of age band (18–24, 25–34, 35–54, 55+), sex, ethnicity (white, Asian, other), index of multiple deprivation quintile^4^ (IMDq), the Government Office Region (GOR) and the respondent’s UTLA’s alcohol-attributable hospital admissions rate and alcohol-related mortality rate. To be clear, we are not saying that the relationship between local characteristics and alcohol consumption is causal, just that they are statistically associated. The admissions rates are taken from the Local Alcohol Profiles for England (LAPE)^5^ and are applied according to age band and sex of the respondent. These can be written as in Eq. 1 and Eq. 2.

1 $$ \Pr {\left(\mathrm{NoDrink}\right)}_{\mathrm{iar}}=f\left(\mathrm{Age}\_\mathrm{Se}{\mathrm{x}}_{iar},\mathrm{Ethnicit}{\mathrm{y}}_{iar},\mathrm{IMD}{\mathrm{q}}_{iar},\mathrm{GO}{\mathrm{R}}_{iar},\mathrm{HE}{\mathrm{S}}_{ar},\mathrm{MOR}{\mathrm{T}}_{ar}\right) $$

2 $$ \Pr {\left(\mathrm{ConsumptionBand}=x\right)}_{iar}=f\left(\mathrm{Age}\_\mathrm{Se}{\mathrm{x}}_{iar},\mathrm{Ethnicit}{\mathrm{y}}_{iar},\mathrm{IMD}{\mathrm{q}}_{iar},\mathrm{GO}{\mathrm{R}}_{iar},\mathrm{HE}{\mathrm{S}}_{ar},\mathrm{MOR}{\mathrm{T}}_{ar}\right) $$

where subscript *iar* denote individual *i* in age-sex group *a* in UTLA *r*. Variables were treated as categorical except for HES_*ar*_ (the rate of alcohol attributable hospital admission episodes per 1000 population) and MORT_*ar*_ (the rate of alcohol attributable mortality per 1000 population), which were modelled as continuous variables. Using a separate logistic regression for abstention from drinking allows the direction of the coefficients to vary. For example, those in areas with high hospital admissions rates may be more likely to abstain, but drink more conditional on not being an abstainer. Such divergent patterns between abstention and heavy consumption are seen in the international literature [5]. These explanatory variables are chosen because they have previously been shown to be significant predictors of alcohol consumption [2] and because there is known population data for each UTLA that can be used to calculate population sizes in the subgroups defined by the combination of age, sex, ethnicity and IMD quintile. For robustness, alternative specifications of the regressions are tested including changing the number of consumption bands (from 6 pre-defined bands to 20 equal bands, i.e. 5% in each band), and removing Government Office Region as a predictor. These make very minor differences to the estimates in terms of mean consumption and are shown in the Appendixes 1, 2 and 3. We also ran the analysis using several different model structures and assessed the models on goodness-of-fit statistics (AIC and BIC) and goodness-of-fit at Government Office Region level mean consumption estimates. Specifically, we investigated using multilevel models and a simultaneous model which estimates abstention and consumption together. However, the multinomial logistic regression with separate abstention provided the best fit and is our preferred model.

Once the regression parameters are estimated, these are applied for each combination of characteristics and UTLAs. For example, we calculate the probability that a male, aged 18–24, of white ethnicity, in IMDq 3, in Sheffield, drinks 11–20 units per week is 19.6%. These are then applied to the known population data for each UTLA. The population data comes from the Office for National Statistics mid-year population estimates for 2013 (ONS, 2013). For example, there are 5196 males aged 18–24 of white ethnicity in IMDq 3 in Sheffield, so we estimate for example that there are 1017 males aged 18–24 of white ethnicity in IMDq 3 in Sheffield drinking 11–20 units per week. This calculation is done for all combinations of characteristics and UTLAs.

These population subgroup estimates by drinker level are then used to create a new survey weight for each individual in the HSE—a survey weight specific to UTLA. This is done by dividing the number in the UTLA population with a set of demographic characteristics and consumption band by the number of respondents with the same set of demographic characteristics and consumption band. This can be written mathematically as:

3 $$ {w}_{idcr}=\frac{N_{dc r}}{n_{dc}} $$

where *w*_*idcr*_ is the weight given to an individual *i* with demographic characteristics *d*, consumption band *c* for UTLA *r*; *N*_*dcr*_ is the number in the population with demographic characteristics *d*, with estimated consumption band *c*, in UTLA *r*; and *n*_*dcr*_ is the number of HSE respondents with demographic characteristics *d*, consumption band *c*. The denominator in Eq. 3 is the same for every UTLA because the number of HSE respondents by subgroup and drinker level does not differ. This now means that we can calculate 151 different weights for each individual HSE respondent—one for each UTLA. This can be used to make the HSE representative for any UTLA, and any statistic of interest on alcohol consumption can be estimated for any UTLA.

## Results

### Regression results

The regression results from the logistic regression for the probability of not drinking, and the multinomial logistic regression for the probability of belonging to each consumption band, are presented in Table 2.

**Table 2:** Regression results for abstaining and of being in consumption bands (1 to 6)

	Abstainer	Mean weekly units of alcohol consumption band					
	(0)	1	2	3	4	5	6
		0 to 10 units	10–20	20–30	30–40	40–50	50+
Sex-age group							
Male 18–24	*(ref)*	*(ref)*	*(ref)*	*(ref)*	*(ref)*	*(ref)*	*(ref)*
Female 18–24	0.407***	*(ref)*	− 0.514***	− 0.533***	− 0.967***	− 0.585*	− 1.017***
	(0.144)		(0.143)	(0.191)	(0.260)	(0.342)	(0.278)
Male 25–34	− 0.323**	*(ref)*	0.018	0.039	− 0.179	− 0.010	− 0.108
	(0.141)		(0.122)	(0.163)	(0.203)	(0.282)	(0.207)
Female 25–34	0.323**	*(ref)*	− 0.620***	− 0.772***	− 1.426***	− 0.983***	− 1.620***
	(0.130)		(0.126)	(0.170)	(0.237)	(0.313)	(0.265)
Male 35–54	− 0.064	*(ref)*	− 0.046	0.359**	0.000	0.382	0.024
	(0.130)		(0.117)	(0.151)	(0.188)	(0.258)	(0.193)
Female 35–54	0.318***	*(ref)*	− 0.511***	− 0.570***	− 1.018***	− 0.656**	− 1.042***
	(0.119)		(0.110)	(0.147)	(0.185)	(0.258)	(0.193)
Male 55+	0.390**	*(ref)*	− 0.274*	0.250	− 0.128	0.211	− 0.232
	(0.157)		(0.145)	(0.184)	(0.241)	(0.308)	(0.239)
Female 55+	1.163***	*(ref)*	− 0.791***	− 0.736***	− 1.399***	− 0.744***	− 1.846***
	(0.117)		(0.110)	(0.146)	(0.189)	(0.256)	(0.213)
IMD quintile							
1 (least deprived)	*(ref)*	*(ref)*	*(ref)*	*(ref)*	*(ref)*	*(ref)*	*(ref)*
2	0.186***	*(ref)*	− 0.057	0.047	− 0.056	− 0.013	− 0.113
	(0.065)		(0.057)	(0.071)	(0.102)	(0.128)	(0.114)
3	0.334***	*(ref)*	− 0.106*	− 0.158**	− 0.234**	0.015	− 0.121
	(0.062)		(0.056)	(0.074)	(0.106)	(0.126)	(0.113)
4	0.615***	*(ref)*	− 0.226***	− 0.144*	− 0.311***	− 0.064	− 0.137
	(0.062)		(0.061)	(0.078)	(0.113)	(0.136)	(0.118)
5 (most deprived)	1.003***	*(ref)*	− 0.384***	− 0.188**	− 0.409***	0.032	− 0.052
	(0.063)		(0.068)	(0.086)	(0.126)	(0.144)	(0.123)
Government Office Region (GOR)							
North East	*(ref)*	*(ref)*	*(ref)*	*(ref)*	*(ref)*	*(ref)*	*(ref)*
North West	0.056	*(ref)*	− 0.053	− 0.136	− 0.416***	0.032	− 0.381***
	(0.078)		(0.084)	(0.105)	(0.146)	(0.178)	(0.138)
Yorkshire and the Humber	0.152*	*(ref)*	− 0.155	− 0.253**	− 0.473***	− 0.074	− 0.562***
	(0.086)		(0.096)	(0.119)	(0.168)	(0.199)	(0.167)
East Midlands	− 0.182*	*(ref)*	− 0.024	− 0.404***	− 0.472***	− 0.271	− 0.464***
	(0.095)		(0.097)	(0.127)	(0.173)	(0.214)	(0.172)
West Midlands	− 0.068	*(ref)*	− 0.077	− 0.258**	− 0.384**	− 0.212	− 0.321**
	(0.087)		(0.093)	(0.117)	(0.161)	(0.202)	(0.156)
East of England	− 0.128	*(ref)*	− 0.303***	− 0.525***	− 0.821***	− 0.605***	− 0.754***
	(0.101)		(0.106)	(0.133)	(0.192)	(0.233)	(0.191)
London	− 0.201	*(ref)*	0.021	− 0.451***	− 0.527**	− 0.805***	− 0.847***
	(0.124)		(0.135)	(0.173)	(0.247)	(0.299)	(0.256)
South East	− 0.116	*(ref)*	− 0.056	− 0.214*	− 0.545***	− 0.278	− 0.539***
	(0.093)		(0.097)	(0.122)	(0.173)	(0.212)	(0.172)
South West	− 0.087	*(ref)*	− 0.108	− 0.287**	− 0.507***	− 0.426**	− 0.787***
	(0.093)		(0.094)	(0.118)	(0.165)	(0.212)	(0.176)
Ethnicity							
White	*(ref)*	*(ref)*	*(ref)*	*(ref)*	*(ref)*	*(ref)*	*(ref)*
Asian	2.600***	*(ref)*	− 0.887***	− 1.098***	− 1.328***	− 1.082***	− 1.542***
	(0.063)		(0.134)	(0.193)	(0.311)	(0.343)	(0.362)
Other	1.436***	*(ref)*	− 0.757***	− 0.936***	− 0.722***	− 0.992***	− 0.644***
	(0.072)		(0.126)	(0.178)	(0.242)	(0.328)	(0.249)
Local characteristics							
Alc-attributable hospital admissions	11.107	*(ref)*	32.382	− 0.832	0.656	49.493	64.317*
	(20.358)		(20.374)	(25.058)	(35.363)	(39.557)	(33.360)
Alc-related mortality	− 501.222	*(ref)*	− 80.800	− 1344.139**	− 876.633	− 2981.573***	− 379.819
	(429.614)		(465.335)	(595.386)	(875.668)	(1016.384)	(860.621)
Constant	− 2.622***	*(ref)*	− 0.672***	− 0.589*	− 0.932**	− 1.433**	− 1.610***
	(0.255)		(0.258)	(0.333)	(0.472)	(0.572)	(0.466)
Observations	24,685	20,422	20,422	20,422	20,422	20,422	20,422

Standard errors in parentheses. Significance: *p < 0.1, **p < 0.05, ***p < 0.01

The results from the logistic regression show that abstention rates are higher in females across all age ranges (a positive coefficient indicates greater likelihood of being an abstainer than for the reference category) and that older males are more likely to abstain than younger males. Those in the most deprived quintile are most likely to abstain, and the relationship between deprivation quintile and abstention probability is monotonic. There is some slight variation across GORs, but the main predictor of abstention is ethnicity, with those of Asian ethnicity most likely to abstain. Those of white ethnicity are least likely to abstain. Neither alcohol attributable hospital admissions nor mortality are significant predictors of abstention.

The results from the multinomial logistic regression for consumption band show that females are less likely to be in the highest consumption bands than males, but unlike abstention, there is no significant difference between older and younger males. The least deprived (people in more affluent areas) are more likely to be in higher consumption bands than those from poorer areas. Unlike abstention, there is large regional variation in consumption bands, with the North East reference category most likely to be in higher consumption bands, followed by the North West. Again, ethnicity is a significant predictor of alcohol consumption, with those of Asian ethnicity much less likely to be in a higher consumption band, even amongst those who drink. The LA-level alcohol attributable hospital admissions rate variable is significantly related to greater probability of being in the highest consumption band and the trend looks somewhat ‘U-shaped’, i.e. areas with higher admissions mean greater chance of being in the lower consumption and higher consumption bands and lesser chance of being in the mid-range consumption bands (though some of these coefficients are not significantly different from zero). In contrast, mortality is negatively and significantly related which may be counter-balancing the coefficient on hospital admissions since these variables are correlated.

### Local Authority variation

**Fig. 2:**
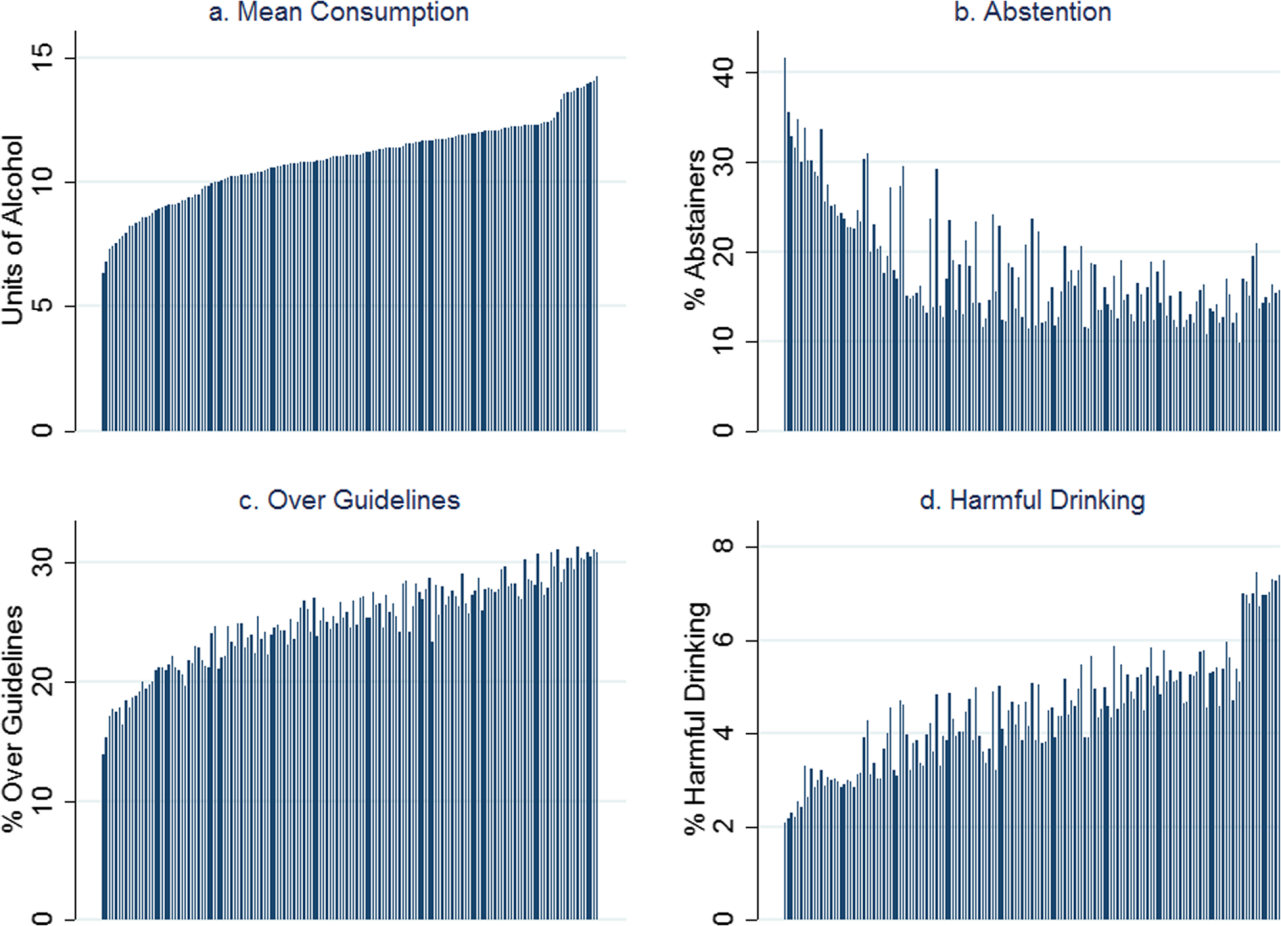
**a**–**d** Alcohol consumption distribution from the Health Survey for England 2011–2013

Four consumption metrics for each UTLA are shown in Fig. 2. There is variation in the estimates of mean weekly consumption across UTLAs, with 2-fold variation between the lowest estimates (around 7 units per week) and the highest (around 14 units per week). Abstention estimates again show large variation across UTLAs, from as low as 11% to as high as 42%, which is likely driven by variation in ethnicity. Because the *x*-axis is sorted by mean consumption estimate, it shows that there is correlation between abstention and mean consumption but that some areas have high abstention and high mean consumption. The estimates for proportion of people drinking over the recent Chief medical Officers’ guidelines of 14 units vary from around 13% of the population to over 30%, and those for drinking harmfully^6^ vary from around 2% to almost 8% of the population, and both are strongly correlated with the mean consumption estimates.

### Comparison with observed data

**Fig. 3:**
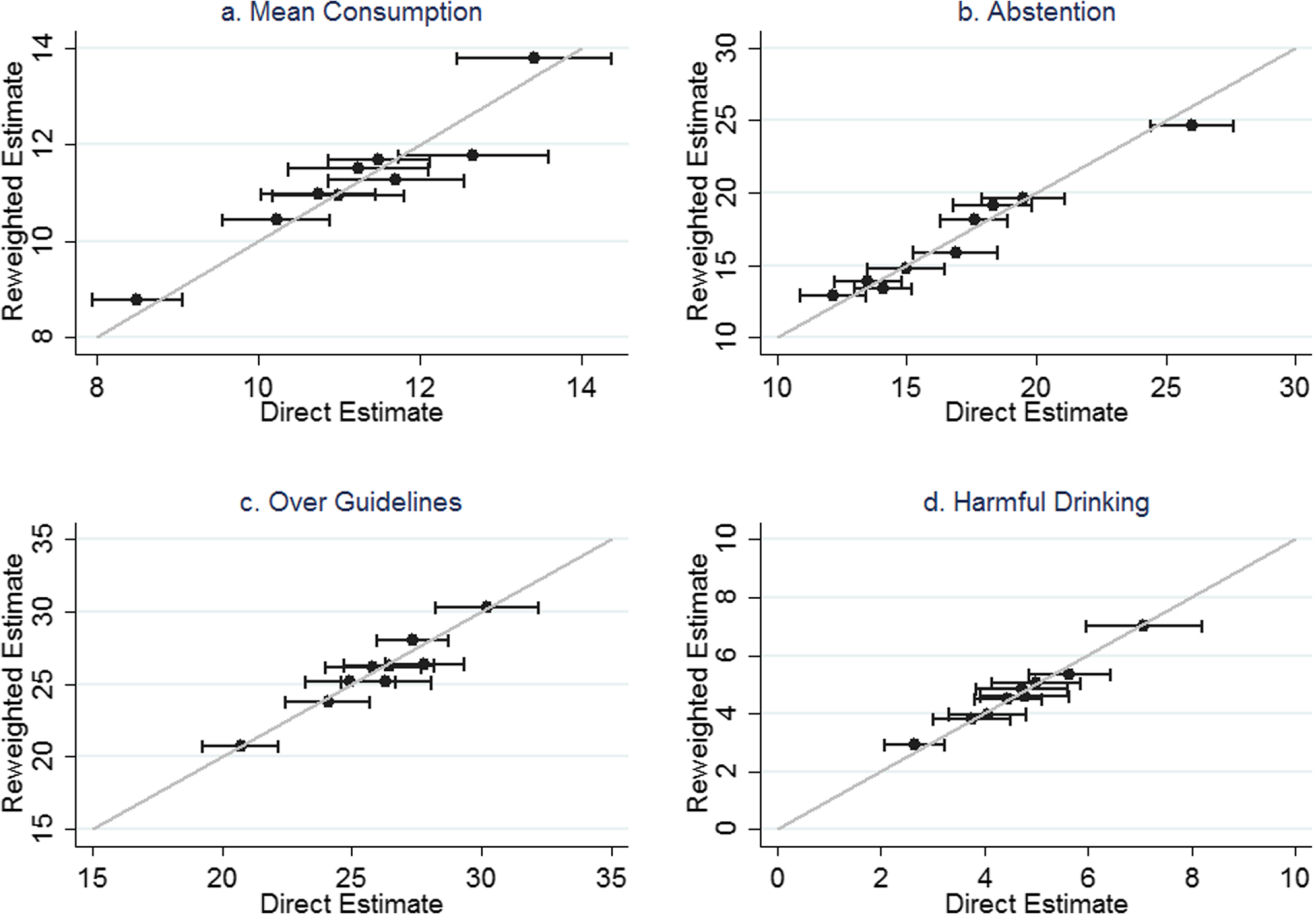
**a**–**d** Comparison between reweighted estimates and HSE data at region level

One method of validation is to compare results generated from the reweighting method with directly measured statistics at GOR level, since the HSE is designed to be representative at this level. Four scatter plots comparing reweighted estimates with direct measures are presented in Fig. 3. The model performs very well at predicting GOR-level estimates with all estimates lying within the 95% confidence intervals calculated from the observed data. The correlation coefficient between reweighted estimates and GOR-level direct measures are 0.95, 0.98, 0.82 and 0.99 for mean consumption, abstention, over guidelines and harmful drinking respectively.

## Discussion

This paper has presented a method of reweighting nationally representative data so that it is representative at the local authority level; in this case, constructing 151 locally representative versions of the Health Survey for England. This was done by estimating the population of each local authority according to age band, sex, ethnicity, index of multiple deprivation and alcohol consumption. Dividing the number of people with these characteristics in each local authority population by the number of respondents with the characteristics in the survey gives a new survey weight. Our findings show substantial variation in estimated alcohol consumption and abstention rates across UTLAs with a 4.5-fold variation in the estimated abstention rates and a 2-fold variation in the estimated mean consumption. Our results are stable across the alternative specifications of statistical models as shown in the Appendixes 1, 2 and 3. Although the results presented in this paper are specific to the British context, the underlying modelling and methods are not. The work could easily be adapted to other countries where local-level explanatory data can be merged into nationally representative survey data.

Local Authorities need to be aware of the variation in estimated drinking volumes, given that there are several policy options, such as licencing decisions and provision of screening and brief interventions, that are decided at the local level. Despite the limitations of the models, the results have clear potential to be used by local decision-makers. The model fit compared to direct estimates at the Government Office Region level is excellent. A potentially useful feature of the reweighting method is that an estimate of the dependent variable (i.e. consumption) can be obtained for any cut-off—so that policymakers could estimate how many people drink above any number of units in their area. Individual areas may also wish to use these estimates for benchmarking and perhaps plan or prioritise services accordingly. The reweighted HSE can be used, in conjunction with other local data sources, to model local policy interventions, and we already have a research project underway to model the potential impact of local minimum unit pricing using an adapted version of the Sheffield Alcohol Policy Model. Given the heterogeneous effects of policies effects across population subgroups, the capability of our new methods to enable local-level modelling of outcomes for subgroups stratified by age, gender and social deprivation is especially important.

There are several limitations to the method presented, as well as some assumptions which will carry through to any modelling work. Perhaps, the most important assumption is that the statistical relationship between the left and right hand side variables is constant across the UTLAs; the effect of being male on consumption for example is assumed to be similar across all UTLAs. However, this is always implicitly the case with survey weights more generally, in that survey respondents are representative of their sample frame. Further to this point, caution is required when looking at variables not included in the analysis, such as, in this case, smoking habits. These wider attributes of the respondents have not been modelled here and may differ by UTLA. The analysis presented here is also not estimated at individual drinking patterns, so analysis by beverage type (e.g. comparing beer consumption by heavy drinkers between UTLAs) would carry large assumptions. That is not to say analysis of this type is not feasible; simply that further work could address this issue through the inclusion of exogenous local data on beverage preferences. Further work could also look at why some regions of England drink more than others, even when controlling for explanatory factors including demographics and hospitalisation rates. This has been noted in the existing literature [2]. More explanatory factors would help improve the explanatory power of the model, which is a limitation of small area estimation generally, as discussed in several papers and reports [16, 22].

This paper has several implications for future-related research. Firstly, more detailed validation against external data would require locally representative data to be collected. This is not easy. Public Health England have conducted surveys in 25 UTLAs to get a measure of local consumption [15]. The reweighted method correlates moderately well, but the sample size of the local surveys is not large enough to be sufficient. Public Health England have also generated small area estimates of “binge” drinking, based on peak daily consumption in the last week. However, these estimates are not comparable with our model which uses usual weekly consumption. Extensions of this work could provide updates when new data becomes available, or look at other health risk factors such as smoking or obesity. Combinations of behaviours, to allow multi-behaviour modelling, could be analysed. Harm risks are particularly acute when individuals have multiple unhealthy behaviours as the risks are multiplicative, and unhealthy behaviours tend to cluster within individuals [3]. Furthermore, the method of reweighting presented in this paper is not unique to either alcohol or small geographical areas and can be applied to a whole host of outcomes, estimates of which are not directly available for small populations. None of our analysis has looked at geography within the UTLA boundary, for example at electoral ward level or even finer geographies that could relate to specific licencing decisions for on-trade or off-trade outlets.

## Conclusion

In conclusion, this paper shows that reweighting nationally representative surveys to make them representative at the local level is possible and finds large variation in alcohol abstention, mean consumption and measures of heavy drinking across UTLAs. The results of our estimation when aggregated up to provide Government Office region estimates align closely with directly observed data. This method could be used in any country where national survey data are available and could be applied to many other outcomes of public health interest to inform local priorities and decisions.

## Figures and Tables

**Fig. 4:**
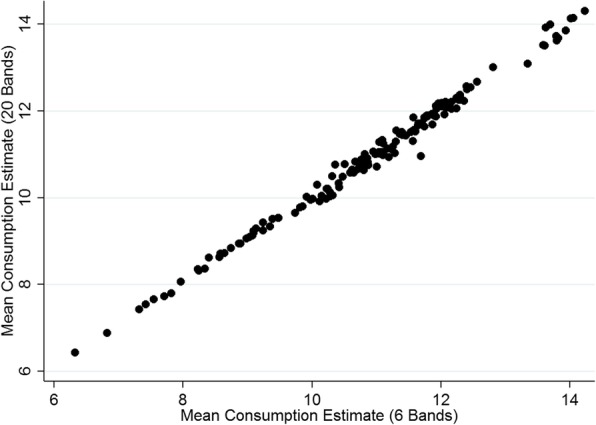
Robustness comparison with 20 consumption bands

**Fig. 5:**
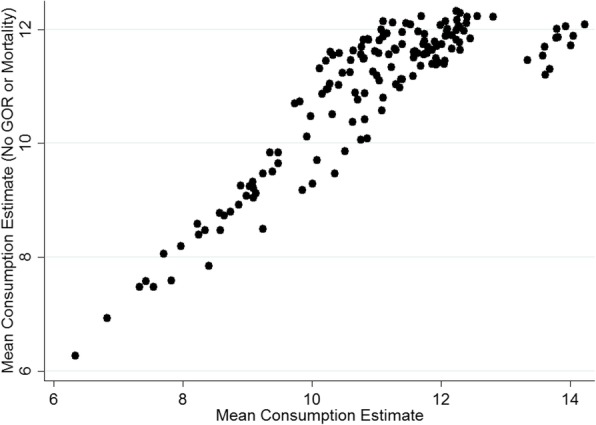
Robustness comparison with variables omitted

**Fig. 6:**
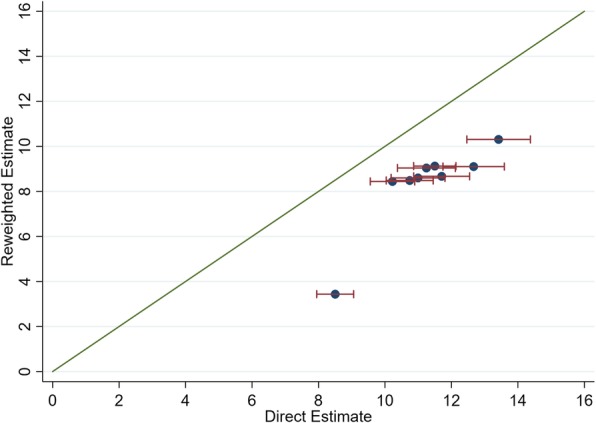
Comparison of multilevel model estimates

**Fig. 7:**
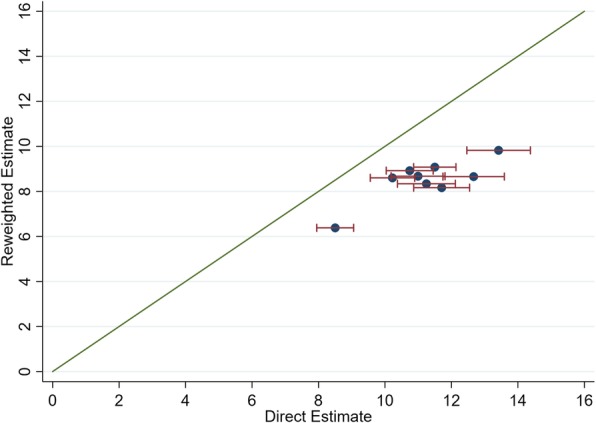
Comparison of single equation model estimates

## Data Availability

We are happy to share our syntax with interested parties. The data will need to be obtained through the UK Data Service due to confidentiality.

## Appendix 1

Robustness checks were carried out. These consisted of testing using a different specification of 20 equally distributed consumption bands, and testing with a regression model that did not include Government Office Region and mortality as explanatory variables. The results are broadly similar to the results presented in the main results section of this paper. Furthermore, excluding GOR and mortality results in worse model fit compared to the direct estimates.

## Appendix 2

We also attempted to fit a regression using a multi-level modelling approach in the form of generalised structural equation modelling. Here we present the regression output and a comparison of predicted and observed mean consumption at Government Office Region level. Although the AIC and BIC of the model are slightly lower than our preferred model specification presented in the paper, the predictions generated from the model do not fit well with observed data. The main difference between the models is in London UTLAs.

**Table 3:** Multilevel model estimates

	Consumption band					
	1	2	3	4	5	6
Sex-age group						
Male 18–24	*(ref)*	*(ref)*	*(ref)*	*(ref)*	*(ref)*	*(ref)*
Female 18–24	*(ref)*	− 0.51***	− 0.52***	− 0.94***	− 0.56*	− 1.01***
		(0.14)	(0.19)	(0.26)	(0.34)	(0.28)
Male 25–34	*(ref)*	0.02	0.04	− 0.19	− 0.02	− 0.12
		(0.12)	(0.16)	(0.20)	(0.28)	(0.21)
Female 25–34	*(ref)*	− 0.62***	− 0.77***	− 1.41***	− 0.98***	− 1.63***
		(0.13)	(0.17)	(0.24)	(0.31)	(0.26)
Male 35–54	*(ref)*	− 0.04	0.37**	− 0.01	0.38	0.02
		(0.12)	(0.15)	(0.19)	(0.26)	(0.19)
Female 35–54	*(ref)*	− 0.50***	− 0.56***	− 1.00***	− 0.65**	− 1.04***
		(0.11)	(0.15)	(0.18)	(0.26)	(0.19)
Male 55+	*(ref)*	− 0.27*	0.25	− 0.17	0.18	− 0.24
		(0.14)	(0.18)	(0.24)	(0.30)	(0.24)
Female 55+	*(ref)*	− 0.78***	− 0.73***	− 1.38***	− 0.74***	− 1.84***
		(0.11)	(0.15)	(0.19)	(0.26)	(0.21)
IMD quintile						
1 (least deprived)	*(ref)*	*(ref)*	*(ref)*	*(ref)*	*(ref)*	*(ref)*
2	*(ref)*	− 0.06	0.02	− 0.06	− 0.05	− 0.14
		(0.06)	(0.07)	(0.10)	(0.13)	(0.11)
3	*(ref)*	− 0.10*	− 0.19**	− 0.24**	− 0.03	− 0.15
		(0.06)	(0.07)	(0.11)	(0.13)	(0.11)
4	*(ref)*	− 0.23***	− 0.19**	− 0.32***	− 0.13	− 0.17
		(0.06)	(0.08)	(0.11)	(0.14)	(0.12)
5 (most deprived)	*(ref)*	− 0.40***	− 0.23***	− 0.42***	− 0.04	− 0.08
		(0.07)	(0.09)	(0.13)	(0.14)	(0.12)
Ethnicity						
White	*(ref)*	*(ref)*	*(ref)*	*(ref)*	*(ref)*	*(ref)*
Asian	*(ref)*	− 0.85***	− 1.10***	− 1.28***	− 1.11***	− 1.52***
		(0.13)	(0.19)	(0.31)	(0.34)	(0.36)
Other	*(ref)*	− 0.75***	− 0.97***	− 0.73***	− 1.07***	− 0.68***
		(0.13)	(0.18)	(0.24)	(0.33)	(0.25)
Local characteristics						
Alc-attributable hospital admissions	*(ref)*	34.28*	1.33	12.94	57.19	68.67**
		(20.16)	(24.80)	(34.05)	(37.72)	(32.24)
Alc-related mortality	*(ref)*	− 173.78	− 337.27	18.14	− 695.60	1248.77*
		(377.08)	(479.48)	(668.24)	(720.45)	(671.42)
UTLA multi-level effect	*(ref)*	1.00	1.29***	1.67***	1.60***	1.63***
		(.)	(0.30)	(0.44)	(0.47)	(0.44)
Constant	*(ref)*	− 0.73***	− 1.28***	− 1.85***	− 2.69***	− 2.82***
		(0.18)	(0.23)	(0.31)	(0.37)	(0.32)
Multi-level variance	0.02**					
	(0.01)					
Observations	20,422	20,422	20,422	20,422	20,422	20,422

Standard errors in parentheses. Significance: **p* < 0.1, ***p* < 0.05, ****p* < 0.01

Log-Likelihood, − 24,065; Pseudo-*R*^2^, N/A; AIC, 48,299; BIC, 48,973

## Appendix 3

In addition to the multilevel model, we fitted a multinomial logistic regression with abstention as its own consumption band (i.e. all drinkers together rather than 2 separate models). Again, the mean consumption estimates at GOR level do not fit as well as our preferred model specification. A scatterplot showing this is presented in Fig. 7.

## Footnotes

1 Throughout this work, the Isles of Scilly are included within Cornwall.

2 For more information on the Health Survey for England, see http://content.digital.nhs.uk/healthsurveyengland

3 For more on this, see Greene and Hensher [9].

4 IMD is a composite measure of deprivation covering 7 domains including income, employment, health, education, housing, crime and the living environment.

5 For more information, see http://www.lape.org.uk/

6 Harmful drinking is classified as drinking more than 35 (50) units per week for females (males) [included in the text].
